# Supplementary material for: Long Term Risks to Neonatal Health from Exposure to War—9 Years Long Survey of Reproductive Health and Contamination by Weapon-Delivered Heavy Metals in Gaza, Palestine
Source: Int J Environ Res Public Health. 2020 Apr 8;17(7):2538. doi: 10.3390/ijerph17072538 (PMC7177220; doi:10.3390/ijerph17072538)
Supplement: Supplementary file 1 [file ijerph-17-02538-s001.zip › Supplementary Materials- File 1.pdf]

SHIFA MATERNITY 2018-19 file number interviewer Initials and date of birth

MOTHER ID mother name mother age father age

parents are cousin=1 relative=2 outside=3

mother telef. residence North=1 Gaza=2 Midlle=3 KanY=4 Rafah=5

Mother preeclampsia 0-no,1-yes Broken Membranes 0-no,1-yes

Delivery: 1- normal, 2- caesarean IVF: 0-no ,1-yes

CHILD gestational age in weeks weigth in grams

sex M male F female single =1 twins=2 more=3

APGAR total score child health : excellent=1 good=2 fair=3 bad=4 dead=5

this child preterm 0-no 1-yes this child birth defect 0-no 1-yes

if birth defect Diagnosis and CD10 class

**Other children**	N	year born	sex	Diagnosis/CD10
previous healthy children				
Previous children birth defect				
Previous children preterm			Alive? Y or N	
Previous children still born			Birth defect? BD<br>Preterm? P	
Previous miscarriages<br><br>Previous child dead after birth		Age at death (months)	BD<br><br>normal<br>BD<br>preterm	Cause of death

MOTHER

Education 0-no school 1-primary 2-econdary 3-diploma 4-master 5-university Mother Occupation presen/previous

chronic diseases 0-none 1-yes which disease Mother diseases during pregnancy 0-none 1-yes which disease

relatives with birth defects and type (ICD10 or diagnosis) 0-none 1-parents 2-brother or sister 3-cousin 4-child of brother 5-child of sister

brothers healthy N

brother's children healthy N

sisters healthy N

sisters children healthy N

FATHER

Education 0-no school 1-primary 2-econdary 3-diploma 4-master 5-university or

Occupation present / previous diseases

relatives with birth defects and type (ICD10 or diagnosis) 0-none 1-parents 2-brother or sister 3-cousin 4-child of brother 5-child of sister

brothers healthy N Father brother's children healthy N

sisters healthy N Father sisters children healthy N

MOTHER where you lived during 2014 attacks? Do you still live there?

Source drinking-cooking water: 1-well 2-pipes 3-delivery 4-bottle

source of food 1-local 2-UNWRA 3-Israel 4-Egypt 5-Turkey

near your house there is 1- open sewage, 2-sewage plants, 3- disposal of garbage , 4-garbage burning in open air, 0- none

your house is near 1-agricultural land? 2-to factories….specify, 0-none Parents use: 1-pesticides 2-insecticide 3-weed killers 4-other chemicals 0- none

was your house hit in attacks in 2014? 0-no answer 1-yes 2- no

where you inside? 0-no answer 1-yes, 2- no

residence attacked with 1-Bombs, 2-air/navy missiles, 3-artillery mortars, 4-incendiary

in 2014 was any house hit next to your residence at the time? 1-yes 0-no

after 2014 attacks did you 1-clean rubble , 2-reuse items from attacked building , 3-rebuilt on site of attacked building, 4-used brick or reused materials to fix/build the house , 0- none

after 2014 bombing-attacks you found objects from weapons 1-in your house, 2-near your house 0-none

during pregnancy has taken 1-painkiller 2-antidepressants 3-Vitamins 4-Folic acid 5- Iron 6-other medicines(specify) 0-none

smoke during pregnancy 0- no, 1-yes

diet in pregnancy 1-excellent 2-good 3-poor

eat fish 0-never 1-once/month 2-twice/month 3-once/week 4-more times/week

eat meat/chicken 0-never, 1-once/month, 2-twice/month 3-once/week 4-more times/week

eat milk products (yogurt, cheeses): 0-never, 1-once/month, 2-twice/month 3-once/week 4-more times/week

eat eggs 0-never, 1-once/month, 2-twice/month 3-once/week 4-more times/week

eats fresh fruit or vegetables 0-never 1-once/ month 2-twice/month 3-once/week 4-more times/week

eat legumes 0-never, 1-once/month 2-twice/month 3-once/week 4-more times/week

consensus x hair samples and x follow up
