# Supplementary material for: Long Term Risks to Neonatal Health from Exposure to War—9 Years Long Survey of Reproductive Health and Contamination by Weapon-Delivered Heavy Metals in Gaza, Palestine
Source: Int J Environ Res Public Health. 2020 Apr 8;17(7):2538. doi: 10.3390/ijerph17072538 (PMC7177220; doi:10.3390/ijerph17072538)
Supplement: Supplementary file 1 [file ijerph-17-02538-s001.zip › Supplementary Matierials- File 2.pdf]

Table S1. Association between phenotypes of singleton newborn and parents characteristics, risky exposures and reproductive history in 2018-2019.

	Birth Defects (N=77)				Preterm Birth (N=305)				Low Birth Weight (N = 113)			
Features	Ca/Nc	OR	*95%CI*	p	Ca/Nc	OR	*95%CI*	p	Ca/Nc	OR	*95%CI*	p
Parents characteristics												
Mother age (year)				0.034				0.506				0.921
< 20	7/477	Ref	-		36/431	Ref	-		13/415	Ref	-	
20-29	44/3008	1.0	*0.45 – 2.2*		196/2783	0.84	*0.58 – 1.2*		72/2698	0.85	*0.47 – 1.6*	
30-39	18/930	1.3	*0.55 – 3.2*		69/851	0.97	*0.64 – 1.5*		23/823	0.89	*0.45 – 1.8*	
≥ 40	6/90	4.5	*1.5 – 13.8*		4/85	0.56	*0.20 – 1.6*		3/82	1.2	*0.33 – 4.2*	
Mother education				0.461				0.360				0.063
No school /primary	15/747	Ref	-		56/681	Ref	-		26/652	Ref	-	
Secondary or more	61/3783	0.80	*0.45 – 1.4*		249/3492	0.87	*0.64 – 1.2*		87/3387	0.64	*0.41 – 1.0*	
Working mother				0.491				0.185				0.265
No	70/4298	Ref	-		285/3962	Ref	-		105/3839	Ref	-	
Yes	5/219	1.4	*0.56 – 3.5*		20/199	1.4	*0.87 – 2.2*		8/188	1.6	*0.75 – 3.2*	
Primiparity				0.871				0.409				0.122
No	52/3098	Ref	-		201/2864	Ref	-		70/2783	Ref	-	
Yes	25/1431	1.0	*0.64 – 1.7*		102/1309	1.1	*0.87 – 1.4*		43/1256	1.4	*0.93 – 2.0*	
Previous children with birth defects				0.600				0.095				n.e.
No	76/4495	Ref	-		299/4143	Ref	-		113/4009	Ref	-	
Yes	1/33	1.8	*0.24 – 13.3*		5/28	2.5	*0.95 – 6.5*		0/28	n.e.	*n.e.*	
Birth defects in the mother's family				0.527				0.061				0.185
No	72/4404	Ref	-		291/4062	Ref	-		112/3931	Ref	-	
Yes	3/123	1.5	*0.46 – 4.8*		14/109	1.8	*1.0 – 3.2*		1/106	0.33	*0.05 – 2.4*	
Father age (year)				0.062				0.922				0.370
< 29	36/2498	Ref	-		170/2299	Ref	-		63/2225	Ref	-	
30-39	25/1602	1.1	*0.65 – 1.8*		110/1476	1.0	*0.79 – 1.3*		42/1425	1.0	*0.70 – 1.5*	
≥ 40	13/397	2.3	*1.2 – 4.3*		25/367	0.92	*0.60 – 1.4*		6/360	0.59	*0.26 – 1.4*	
Father education				0.684				0.093				0.161
No school/primary	21/1189	Ref	-		93/1086	Ref	-		36/1044	Ref	-	
Secondary or more	53/3338	0.90	*0.54 – 1.5*		212/3085	0.80	*0.62 – 1.0*		77/2993	0.75	*0.50 – 1.1*	
Father chronic diseases				0.061				0.685				n.e.
No	72/4507	Ref	-		303/4152	Ref	-		113/4018	Ref	-	
Yes	2/22	5.7	*1.3- 24.7*		2/20	1.4	*0.32 – 5.9*		0/2	n.e.	*n.e.*	
Birth defects in the father family				0.199				0.746				0.531
No	70/4407	Ref	-		296/4061	Ref	-		111/3929	Ref	-	
Yes	4/120	2.1	*0.75 – 5.8*		9/110	1.1	*0.56 – 2.2*		2/108	0.66	*0.16 – 2.7*	
Parents consanguineity				0.283				0.149				0.964
No	38/2732	Ref	-		170/2528	Ref	-		68/2448	Ref	-	
Cousin	13/660	1.4	*0.75 – 2.7*		44/608	1.1	*0.76 – 1.5*		16/588	0.98	*0.56 – 1.7*	
Relative	23/1122	1.5	*0.87 – 2.5*		90/1024	1.3	*1.0 – 1.7*		29/990	1.1	*0.68 – 1.6*	

Delivery features												
Infant sex				0.441				0.024				0.003
Male	36/2339	Ref	-		177/2144	Ref	-		41/2095	Ref	-	
Female	40/2174	1.2	*0.76 – 1.9*		127/2015	0.76	*0.60 – 0.97*		68/1934	1.8	*1.2 – 2.7*	
Gestational diabetes				0.276				0.082				0.043
No	73/4292	Ref	-		282/3959	Ref	-		102/3836	Ref	-	
Yes	2/237	0.50	*0.12- 2.0*		23/213	1.5	*0.97 – 2.4*		11/202	2.0	*1.1 – 3.9*	
Type of delivery				0.218				< 0.001				< 0.001
Vaginal	56/3562	Ref	-		213/3304	Ref	-		70/3216	Ref	-	
Cesarean	21/964	1.4	*0.83 – 2.3*		93/864	1.7	*1.3 – 2.2*		43/818	2.4	*1.6 – 3.6*	
In vitro fertilization				0.734				0.214				0.092
No	76/4493	Ref	-		301/4140	Ref	-		110/4009	Ref	-	
Yes	1/41	1.4	*0.20 – 10.6*		5/36	1.9	*0.74 – 4.9*		3/33	3.3	*1.0 – 11.0*	
Residence features												
Residence area				0.584				< 0.001				0.158
North	11/669	Ref	-		48/616	Ref	-		10/603	Ref	-	
Gaza	62/3723	1.0	*0.54 – 1.9*		229/3447	0.85	*0.62 – 1.2*		100/3330	1.8	*0.94 – 3.5*	
Middle	4/134	1.8	*0.57 – 5.8*		27/107	3.2	*1.9 – 5.4*		3/103	1.8	*0.48 – 6.5*	
House hit in 2014				0.853				0.007				0.919
No	57/3513	Ref	-		216/3254	Ref	-		89/3150	Ref	-	
Yes	14/912	0.95	*0.52 – 1.7*		80/825	1.5	*1.1 – 1.9*		22/798	0.98	*0.61 – 1.6*	
Nearby house hit in 2014				0.004				0.148				0.159
No	47/3583	Ref	-		230/3305	Ref	-		96/3191	Ref	-	
Yes	25/903	2.1	*1.3 – 3.4*		71/829	1.2	*0.93 – 1.6*		17/810	0.70	*0.41 – 1.2*	
Rubble use after 2014				0.483				0.143				0.482
No	25/1708	Ref	-		125/1533	Ref	-		45/1476	Ref	-	
Yes	49/2818	1.2	*0.73 – 1.9*		180/2637	0.84	*0.66 – 1.1*		68/2560	0.87	*0.59 – 1.3*	
House near a sewage				0.388				0.313				0.280
No	66/4165	Ref	-		276/3841	Ref	-		107/3714	Ref	-	
Yes	8/359	1.4	*0.67 – 3.0*		29/327	1.2	*0.83 – 1.8*		6/320	0.65	*0.28 – 1.5*	
House near a garbage				0.164				0.236				0.671
No	73/4336	Ref	-		296/3989	Ref	-		109/3859	Ref	-	
Yes	1/188	0.32	*0.04 – 2.3*		9/179	0.68	*0.34 – 1.3*		4/175	0.81	*0.29 – 2.2*	
House near agricultural activities				0.062				0.055				0.353
No	65/3600	Ref	-		229/3327	Ref	-		94/3217	Ref	-	
Yes	9/926	0.54	*0.27 – 1.1*		76/843	1.3	*1.0 – 1.7*		19/819	0.79	*0.48 – 1.3*	
House near factories				< 0.001				0.749				n.e.
No	66/4411	Ref	-		298/4062	Ref	-		113/3928	Ref	-	
Yes	8/115	4.6	*2.2 – 9.9*		7/108	0.88	*0.41 – 1.9*		0/108	n.e.	*n.e.*	

Lifestyle exposures												
Folic acid during pregnancy				n.e.				n.e.				n.e.
No	0/12	Ref	-		0/12	Ref	-		0/12	Ref	-	
Yes	71/4299	n.e.	*n.e.*		297/3955	n.e.	*n.e.*		105/3830	n.e.	*n.e.*	
Mother fish consumption				0.479				0.317				0.076
No	13/659	Ref	-		50/595	Ref	-		10/582	Ref	-	
Yes	61/3866	0.80	*0.44 – 1.5*		255/3574	0.85	*0.62 – 1.2*		103/3453	1.7	*0.90 – 3.3*	
Parents use of fertilizers				0.386				0.048				0.699
No	60/3879	Ref	-		250/3586	Ref	-		96/3473	Ref	-	
Yes	13/638	1.3	*0.72 – 2.4*		55/575	1.4	*1.0 – 1.9*		17/554	1.1	*0.66 – 1.9*	

Ca/Nc = Cases / Non-cases; n.e. = not evaluable.
